# Hemostasis in elderly patients with human immunodeficiency virus (HIV) infection—Cross-sectional study

**DOI:** 10.1371/journal.pone.0227763

**Published:** 2020-02-12

**Authors:** Marilza Campos de Magalhães, Juan Camilo Sánchez-Arcila, Ana Carolina de Brito Lyra, Luiz Felipe Boufleur Long, Isabelle Vasconcellos de Souza, Fernando Raphael de Almeida Ferry, Adilson José de Almeida, Soniza Vieira Alves-Leon

**Affiliations:** 1 Gaffrée and Guinle University Hospital, Postgraduate Program in Neuroscience / Neurology, Federal University of Rio de Janeiro State—UNIRIO, Rio de Janeiro, RJ, Brazil; 2 Viral Immunology Laboratory, Oswaldo Cruz Institute (FIOCRUZ), Rio de Janeiro, RJ, Brazil; University of California Irvine, UNITED STATES

## Abstract

**Introduction**: Aging and chronic HIV infection are clinical conditions that share the states of inflammation and hypercoagulability. The life expectancy of the world population has increased in the last decades, bringing as complications the occurrence of diseases that undergoing metabolic, bone, cardiological, vascular and neurological alterations. HIV-infected patients experience these changes early and are living longer due to the success of antiretroviral therapy. **The objectives** of this study was to evaluate some changes in the plasma hemostatic profile of 115 HIV-reactive elderly individuals over 60 years old in the chronic phase of infection, and compare with 88 healthy uninfected elderly individuals. Plasma determinations of D-dimers, Fibrinogen, von Willebrand Factor, Antithrombin, Prothrombin Time, Activated Partial Thromboplastin Time, and platelet count were performed. In the HIV-reactive group, these variables were analyzed according to viral load, protease inhibitor use and CD4^+^ T lymphocyte values. After adjusted values for age and sex, **the results** showed higher levels of Antithrombin (103%; 88%, *p* = 0.0001) and Prothrombin Time activities (92.4%; 88.2%, p = 0.019) in the HIV group compared to the control group. We observed higher values of Fibrinogen in protease inhibitor users in both the male (*p* = 0.043) and female (*p* = 0.004) groups, and in the female HIV group with detected viral load (p = 0.015). The male HIV group with a CD4^+^ count> 400 cells / mm^3^ presented higher von Willebrand Factor values (p = 0.036). D-Dimers had higher values in the older age groups (p = 0.003; p = 0.042, respectively). Conclusion: Our results suggest that the elderly with chronic HIV infection with few comorbidities had a better hemostatic profile than negative control group, reflecting the success of treatment. Protease inhibitor use and age punctually altered this profile.

## Introduction

Patients with HIV infection are living longer. The survival curve is near to the uninfected population, thanks to antiretroviral therapy (ART), which turned this disease into silent, chronic and cumulative as predicted in early 2000 [1–3]. Currently, non-silent complications related to the treatment itself have emerged, such as dyslipidemia, in metabolic, bone, cardiovascular, thrombotic and neurodegenerative diseases [4–9]. A considerable proportion of patients who acquired HIV at age thirty-forty and who have benefited from the post-ART era are now sixty-year-old, composing the geriatric population with HIV [10]. Despite control of HIV infection, these patients persist with a subclinical inflammatory state due to changes in the immune system by latent infections such as cytomegalovirus and microbial translocation [11,12]. In addition, it is possible to observe endothelial and immune microvascular dysfunctions, hemostasis activations and fibrinolysis,among others [13–16]. Chronic infection of HIV promotes changes in hemostasis and coagulation inconsequence of persistent systemic immune activation, micro- and macro-vascular disease, and, potentially, impaired hepatic synthesis of coagulation factors [17]. Futhermore, in these patients, the aging process starts early compared to the general population, due to the increase of complicating factors and other diseases [18–20]. Markers of biological and cellular aging are common in HIV-reactive patients [21]. They affect lymphocytes and monocytes, increase inflammatory proteins and, when activated by the inflammatory response, express in their cytoplasmic membranes the Tissue Factor (TF), initiating coagulation [22–26].Studies started before and after ART, when compared to others in the geriatric area, confirm the common pre-thrombotic state among the two groups [27,28]. This pre-thrombotic state is usually associated with comorbidities and severity of infection [29], making the association a risk factor for mortality and for the survival rate of these patients not yet the same as the general population [10,24,30–33].

Hemostasis alterations have been investigated in various studies [34,35] on treated infection and aging. Hemostasis alterations in cardiovascular and neurological diseasesare the most frequently observed [7,25,36].The actions in the different phases of hemostasis of coagulation factors may be observed with von Willebrand Factor (vWF), Fibrinogen (Fg), D-dimers (DD), Antithrombin (AT).Fg is a procoagulant protein in primary hemostatic reactions, blood coagulation, interferes with plasma viscosity and one of the most studied [37,38]. Its increase is observed both during natural aging and in patients reactive to HIV, as well as in situations of cellular and tissue stress. Thus, it is considered a biomarker of risk in thrombotic disease [27,28,31,35,39,40]. D-D peptides are products of fibrinolytic activity and there is currently contrasting information about their presentation in aging. This may be due to the likelihood of endothelial cell dysfunction during plasminogen activator release [27,34,41–45]. Classically, they are inflammatory biomarkers, risk factors for cardiovascular disease [35,42,46,47]. However, in the acute form of the disease, D-D are seen as relevant markers for disease progression and a risk factor for mortality [48,49]. AT is an inhibitory serine protein with natural physiological anticoagulant function and anti-inflammatory activity, regulating cell migration and antiviral control [50–53]. The main role of AT is the neutralization of thrombin, forming a thrombin-antithrombin complex, preventing its action on coagulation. The increase in AT can be observed in studies related to aging, menopause and general infections [54]. At menopause, it is associated with protective effects against the rise of procoagulant factors that tend to occur in these situations. vWF is a protein produced by endothelial cells and expressed by these cells when activated. It acts on primary hemostasis, interacting with platelets and coagulation as a transport to the Factor VIII molecule. In aging and infections, it has functioned as a biological parameter to assess disease risk or progression [27,43,49,55].

Most studies involving hemostasis in HIV infection have focused on younger populations or without an appropriate age range, or have cohorts with co-infections and other comorbidities. Studies in this area with elderly patients with chronic infection without other comorbidities are rare. In this context, we aimed to evaluate some hemostasis parameters in this population compared to non-reactive and demographic variables.

## Material and method

The study has a cross-sectional design. Subjects from the HIV-reactive study group (SG) were recruited from 2013–2015 from a sub-cohort of outpatients undergoing treatment in the Immunology / AIDS Sector of the Gaffrée e Guinle Hospital of the Federal University of Rio de Janeiro State where they held regular consultations. All had been on antiretroviral treatment for at least two years. The non-reactive HIV control group (CG) consisted of individuals recruited from the Social Age Support Group Renascer, as well as hospital staff, using the same evaluation and selection process.

Study group (SG) participants were recruited on the following criteria: age 60 and older, both sexes, infected with HIV in the chronic phase. Exclusion criteria for both groups were: presence of other viral infections, infectious or inflammatory processes or surgery less than 30 days old, without the use of anti-inflammatory drugs, without cancer or autoimmune diseases, absence of personal or family history of recurrent thrombosis and bleeding.

The study was approved by the Department of Teaching and Research and the Research Ethics Committee of the Gaffrée e Guinle University Hospital of the FederalUniversity of Rio de Janeiro State,CAAE: 10681613.4.0000.5258 on March 14, 2013. All subjects were invited to participate voluntarily and included in the research after signing the Informed Consent Form, according to Resolution No. 466/2012 of the National Health Council—Ministry of Health, Brazil.

The participants' epidemiological, clinical and laboratory data were obtained from medical records. After admission to the study, a 10 ml blood sample in 3.8% sodium citrate solution and E.D.T.A. was colleted to obtain plasma and then perform coagulation, viral load, and CD4 T-cell count tests. The material was immediately centrifuged, aliquoted into Eppendorf tubes and stored at -70° C for further analysis. Data and biological material were collected from 2014-2015.The obtained results were filed in an Excel spreadsheet for further analysis.

The choice of biological variables was based on observations already established in the literature, both in the context of HIV infection and aging. They were considered due to their performance in different areas of hemostasis and inflammation: routine coagulation test such as Prothrombin Time (TP / INR) and Activated Partial Thromboplastin Time (aPTT); evaluation of primary hemostasis and blood coagulation by Fibrinogen (Fg) and von Willebrand Factor (vWF) dosage; for fibrinolysis, dosage of D-Dimeros (D-D) and, in contrast to coagulation, Antithrombin (AT).

During the pre-inclusion phase, the following laboratory tests were performed on all study subjects: blood count, PT / INR and aPTT; serum biochemical evaluation including liver enzymes, lipid profile, glucose, urea, creatinine (automated tests standardized by the HUGG Central Laboratory). Serum antibodies (IgG / IgM) to HIV, toxoplasmosis, HTLV-I / II, Cytomegalovirus, Epstein-Barr, VDRL, HCV, HBS antigen, anti-HBS (Hospital Central Laboratory).

The following tests were performed for HIV-reactive individuals: CD4^+^ and CD8^+^ T lymphocyte count by BD FACSCount® Controls reagent flow cytometry (BD Biosciences Immunocytometry Systems, San Jose, California, United States of America, [USA]); Determination of HIV viral load by Abbott Real Time HIV-1 (NucliSens® HIV-1 QT, Biomérieux, Boxtel, The Netherlands). The tests were conducted at the Immunology and AIDS / Research Laboratory/ Hospital University Gafrée Guinle.

In all study participants the following analyzes of hemostatic factors were performed following the manufacturers instructions:

D-Dimers: immunoturbidimetry method using LiaTest (Stago Diagnostic, France). Reference values: less than 550.0 ng / mL FEU.

Antithrombin: chromogenic method, (Stachrom® ATIII, Diagnostica Stago, France). Reference values: 83–128% activity.

Von Willebrand factor: latex immunoturbidimetry method, (STA®—Liatest® VWF: A, Diagnostica Stago, France). Benchmarks: 48–239% of activity.

Activated Partial Thromboplastin Time (photo-optic detection in automated coagulometer (ELITE PRO ACL System using SynthASil, HemosIL® Reagent, Instrumentation Laboratory, USA) Reference Values: 25.4–38.4 seconds).

Prothrombin Time / INR (photo-optic detection in an ELITEPRO ACL automated coagulation system using RecombiPlasTin 2G HemosIL® Intrumentation Laboratory, U.S.A. Reference Values: 9.1–12.1 seconds or 70–100% activity.

Fibrinogen (photo-optic detection on the automated ELITEPRO ACL System coagulometer using RecombiPlasTin 2G HemosIL®, Instrumentation Laboratory, U.S.A). Reference Values: 310–430 mg / dl.

### Statistical analysis

The results presented in the tables and values of the text variables were expressed as frequencies, mean, standard deviation or median (interquartile range [IQR]: 25% - 75% quantile). To compare frequencies between groups, the Yates-corrected chi-square test was used. We used the Grubb test to detect outliers and the Kolmogorov-Smirnov test to assess the normality of the data. To compare the values between the two groups, we used an unpaired t-test for variables with normal distribution or nonparametric Mann-Whitney U test for variables without normal distribution; and *post hoc* Kruskal Wallis, a Dunn’s test was used to compare three or more groups. We used generalized linear regression (GML) models to compare the values of hemostasis variables between HIV-reactive and healthy individuals. In order to study the influence of Sex and Age as confounding factors, we incorporated these variables into a new GLM and then calculated new adjusted confidence intervals (CI) of 5% to 95% and adjusted *p* values for these models were calculated for control variables by sex and age. We used False Discovery Rate adjustment for multiple comparisons using the Benjamini-Hochberg approach (1995). P values <0.05% were considered significant. Statistical analysis was performed using the Graphpad Prism version 6.0 statistical package or the R computational environment software version 3.4.3.

## Results

In 2015, three hundred and twenty-five patients aged 60 and over were undergoing treatment at Gaffrée and Guinle University Hospital. To form the SG and CG, 293 individuals were interviewed. Of this total, ninety participants (24.42%) were excluded for technical reasons, exclusion criteria and for not accepting to participate. Two hundred and three (203) subjects were selected, 115 for the SG and 88 for the CG. Female SG was composed of 63 participants (54.80%) and male by 52 (45.20%). The female CG group consisted of 64 participants (72.70%) and the male group by 24 (27.30%). The median age between the groups was 66.0 (10.5; 60.0–80.0) years for the EG and 71.0 (13.5; 60–87) for the CG. Epidemiological data from both groups showed a higher proportion of men in the HIV ^+^ group (p = 0.008) and age (p = 0.0001).

We did not observe differences in leukocyte, lymphocyte and platelet values between groups. Considering the lipid profile, HIV ^+^ individuals had a higher frequency of high cholesterol> 200 mg / dL (p = 0.049); Triglycerides> 130 mg / dL, (p = 0.0002); and LDL> 130, (p = 0.0297). We did not observe differences in hypertension or diabetes prevalence between CG and SG. On the other hand, the HIV group had a higher frequency of smokers (p = 0.018) (Table 1).

**Table 1:** Epidemiological, biochemical and comorbidities data of elderly HIV patientes and control group.

	Control (n = 88)	HIV+ (n = 115)	*P-valor*
***Sex N(%)***			
Male	25(28.4)	91(55.7)	***<0*.*0001***
Female	63(71.6)	64(41.3)	
***Etnia N(%)***			
White	50(56.8)	67(58.3)	0.63
Afro-descendent	26(29.6)	29(25.2)	
Black	12(13.6)	19(16.5)	
***Celleular count (/mm***^***3***^***)***			
CD4^+^	NM	651(421;422–843)	NC
CD8^+^	NM	812(545.5;609–1154.5)	NC
Leucocytes	5.950(2095;4900–6995)	6045(2840;4627.5–7467.5)	0.67
Lymphocytes	1930(777;1513–2290)	2010(997.5;1555–2552.5)	0.16
Platelets	240(85.75;188.5–274.25)	212(79;174–253)	0.16
***Lipidic profile (%)***			
Cholesterol >200 (mg/L)	40.9	62.6	***0*.*05***
Triglycerides>150 (mg/L)	34.1	60.9	***<0*.*001***
HDL> 85 (mg/L)	1.1	3.5	0.65
LDL>130 (mg/L)	34.1	47.0	***0*.*03***
***Comorbidities (%)***			
Hypertension	62.5	54.8	0.38
Diabetes	21.6	22.6	0.99
Smoker	10.2	23.5	***0*.*02***

For frequency comparison, a chi—squared test with Yates-correction was used. For continuous variables we used an unpaired t-test with normal distribution or unpaired nonparametric Mann-Whitney test for variables without normal distribution. Abbreviations: HDL = high density lipoprotein; LDL = lower density lipoprotein; NM = no measured; NC = no calculated.

To address these differences, a logistic regression analysis was performed for each hemostasis variable, controlling for age and gender. We noted that PT was higher in HIV^+^ patients 92.4 (15.7;84.3–100) when compared to Controls 88.2(11.9;82–93.9)(p = 0,02). AT was higher in HIV^+^ patients 103 (24;92–116) when compared to Controls 88(15.25;80–95.25) (p<0,0001) (Table 2).

**Table 2:** Biological parameters of hemostasis in elderly HIV reactive patients and control group.

Variable	Group		Estimate	Std. Error	P-value	CI [5% - 95%]	Adjusted P-value*	Adjusted CI [5% - 95%]
Median (IQR; Quantil 25%-Quantil 75%)	HIV+ (n = 115)	Control (n = 88)						
**aPTT (seg)**	28.1(4.4;26–30.4)	28.6(4.15;26.8–30.95)	-0.169	0.482	0.726	[-0.97 * 0.63]	0.78	[-1.04–0.74]
**PT (% activity)**	92.4(15.7;84.3–100)	88.2(11.9;82–93.9)	0.052	0.017	0.002	[0.02 * 0.08]	***0*.*02***	[0.01–0.07]
**INR**	1.04(0.1;1–1.1)	1.07(0.08;1.04–1.12)	-0.029	0.010	0.004	[-0.05 * -0.01]	***0*.*027***	[-0.04 - -0.01]
**Fg (mg/dL)**	390(154.5;322–476.5)	432(131;379.5–510.5)	-0.113	0.049	0.021	[-0.19 * -0.03]	0.318	[-0.14–0.03]
**D-D (ng/mL FEU)**	266(330;167–497)	391(524.25;222.75–747	-0.236	0.132	0.075	[-0.50 * 0.02]	0.668	[-0.17–0.29]
**vWF (% activity)**	145(70;113–183)	145.5(54.5;115–169.5)	-0.145	0.383	0.705	[-0.90 * 0.61]	0.85	[-0.63–0.79]
**AT (% activity)**	103(24;92–116)	88(15.25;80–95.25)	0.151	0.022	***0*.*0001***	[0.11 * 0.19]	***0*.*0001***	[0.11–0.19]

*To calculate the logistic regression models, each variable was evaluated in the model in relationship with the variable Group (Variable ~ Group). For each variable model, it was calculated P-values and the 5%-95% Confidence interval (CI). Values highlighted in bold represented P-values < 0.05. Considering the potential impact of Sex and Age as confounding factors, we calculated a logistic regression Variable ~ Group + Age + Sex. From these models, were calculated adjusted P values and Confidence intervals. Abbreviations: aPTT = activated partial thromboplastin time; PT = Prothrombin time; INR = International normalized ratio; Fg = Fibrinogen; vWF = vonWillebrand Factor; D-D = D-dimers; AT = Antithrombin.

Subsequently, we evaluated separately in HIV-reactive individuals hemostatic marker values, considering the use of protease inhibitors, viral load (> 40 RNA copies / mL = detectable; < 40 RNA copies / mL = undetectable) and CD4^+^ T cell count. / mm^3^. Eighty-eight subjects (76.52%) had CD4^+^ T lymphocyte count ≥400 / mm^3^; 53 (46.08%) were on protease inhibitor use and 97 (84.34%) had undetectable viral load. To avoid the influence of variable sex as a confounding factor, hemostatic values were separately evaluated in the male and female groups. The proportional differences between them were not statistically significant for the groups during CD4^+^ (p = 0.926), viral load (*p* = 0.199) and PI (*p* = 0.062) analyzes. Significant differences in Fibrinogen were observed in the male (*p* = 0.043) and female (*p* = 0.004) groups using PI compared to those who did not use and in the aPTT values in women using PI (*p* = 0.014) (Table 3).Regarding viral load there was only significant difference in vWF values (*p* = 0.015) in the female group with viral load detected (Table 4). We observed in the TCD4^+^ cell level evaluation a significant difference in the male group with cellularity above 400 / mm^3^ (*p* = 0.036) in the comparison of vWF (Table 5).

**Table 3:** Biological parameters of hemostasis in elderly HIV patients in relation to use of protese inhibitors, by sex.

	Male n = 52(45.20%)			Female n = 63(54.80%)		
*Protease Inhibitor use*						
Variable	Yes	No	*P-valor*	Yes	No	*P-valor*
	n = 19 (35.85%)	n = 33 (53.23%)		n = 34 (64.15%)	n = 29 (46.77%)	
**aPTT**	29.9(8.1;26.2–34.3)	28.2(3.93;25.9–29.82)	0.16	***29*.*15(4*.*85;26*.*75–31*.*6)***	***27(3*.*1;25*.*7–28*.*8)***	***0*.*014***
**PT**	92.4(20.4;85.6–106)	86.95(12.3;83.1–95.4)	0.21	95.4(19.88;82.9–102.78)	94.65(14;89.5–103.5)	0.86
**INR**	1.05(0.12;0.97–1.09)	1.08(0.09;1.02–1.11)	0.24	1.02(0.12;0.98–1.1)	1.03(0.09;0.98–1.07)	0.77
**Fg**	***415(200;333–533)***	***339(159;255–414)***	***0*.*043***	***467(197;375*.*5–572*.*5)***	***357(131*.*5;310–441*.*5)***	***0*.*004***
**D-D**	192(220.5;155–375.5)	191(325;135–460)	0.96	276.5(231.25;207.75–439)	390(536;204–740)	0.49
**vWF**	180(100;116–216)	145(49;120–169)	0.66	147(75.25;113.75–189)	140(80;99–179)	0.51
**AT**	97(25;93–118)	92(18.25;87.75–106)	0.17	105(22;94.5–116.5)	109.5(17;100–117)	0.37

Variable values are represented as median (IQR; minimum-maximum values). To calculate the differences between the groups, we used the T test for normal distribution variables or the Mann-Whitney U test for variables without normal distribution. Abbreviations: TTPa = activated partial thromboplastin time; TP = prothrombin time; INR = international normal ratio; Fg = Fibrinogen; FvW = von Willebrand factor; D-D = D-dimers; AT = Antithrombin; D = detected; ND = not detected. Quantification values: aPTT (seconds); PT (% of activity); INR (ratio) Fg (mg / dL); vWF (% of activity); D-D- (ng / ml FEU); AT (% of activity).

**Table 4:** Biological parameters of hemostasis in elderly HIV patients in relation to viral load, by sex.

	Male n = 52(45.20%)			Female n = 63(54.80%)		
*Viral Load*						
variáble	D	ND	*P-valor*	D	ND	*P-valor*
	n = 6 (31.58%)	n = 47 (48.45%)		n = 13 (68.42%)	n = 50 (51.55%)	
**aPTT**	28.9(3.5;26.48–29.98)	28.2(5;26.2–31.2)	0.89	26.6(1.9;25.9–27.8)	28.2(3.68;26.55–30.23)	0.13
**PT**	86.3(14.15;85.6–99.75)	89.5(15.5;83.1–98.6)	0.89	97.7(11.6;91.68–103.28)	93.9(19.38;84.12–103.5)	0.25
**INR**	1.08(0.08;1.01–1.09)	1.06(0.09;1.01–1.1)	0.9	1.01(0.06;0.98–1.04)	1.03(0.11;0.98–1.1)	0.24
**Fg**	336(30;331.5–361.5)	386.5(146.75;298.75–445.5)	0.83	460(149.25;351.25500.5)	394.5(187.75;346.5–534.25)	0.6
**D-D**	357(67;300–367)	191(314.5;136.5–451)	0.74	350.5(328.5;253.5–582)	334(372;195–567)	0.47
**vWF**	163.5(64.5;131.25–195.75)	145.5(66.5;119–185.5)	1	***172*.*5(48*.*5;150–198*.*5)***	***130(87*.*5;88*.*5–176)***	***0*.*015***
**AT**	94(1;93.5–94.5)	93(18;90–108)	0.94	98.5(15.5;95.75111.25)	106(22;96–118)	0.17

Variable values are represented as median (IQR; minimum-maximum values). To calculate the differences between the groups, we used the T test for normal distribution variables or the Mann-Whitney U test for variables without normal distribution. Abbreviations: TTPa = activated partial thromboplastin time; TP = prothrombin time; INR = international normal ratio; Fg = Fibrinogen; FvW = von Willebrand factor; D-D = D-dimers; AT = Antithrombin; D = detected; ND = not detected. Quantification values: aPTT (seconds); PT (% of activity); INR (ratio) Fg (mg / dL); vWF (% of activity); D-D- (ng / ml FEU); AT (% of activity).

**Table 5:** Biological parameters of hemostasis in elderly HIV patients in relation to TCD4^+^ cellular count, by sex.

	Male n = 52(45.20%)			Female n = 63(54.80%)		
*Células T CD4*^*+*^						
Variable	< 400 Cells / mm^3^	> 400 Cells / mm^3^	*P-valor*	< 400 Cells / mm^3^	> 400 Cells / mm^3^	*P-valor*
	n = 12 (44.44%)	n = 40 (45.44%)		n = 15 (55.56%)	n = 48 (54.55%)	
**aPTT**	28.2(3.5;26.72–30.23)	28.05(3.88;26.1–29.98)	0.81	28.4(5;26.2–31.2)	26(1.95;25.75–27.7)	0.14
**PT**	95.4(17;87–104)	85.6(16.07;83.65–99.72)	0.67	89.5(14.3;84.3–98.6)	93.9(14.8;86.9–101.7)	0.98
**INR**	1.02(0.1;0.98–1.08)	1.09(0.1;1.01–1.1)	0.68	1.06(0.09;1.01–1.1)	1.04(0.09;0.99–1.08)	0.96
**Fg**	409(205;333–538)	345.5(92.25;331.5–423.75)	0.74	386.5(132.5;298.75–431.25)	426(106;372–478)	0.82
**D-D**	380(477;213.5–690.5)	245.5(212.25;159–371.25)	0.8	*191*.*5(311*.*75;134*.*75–446*.*5)*	*247(113;160*.*5–273*.*5)*	*0*.*06*
**FvW**	***140(82;99–181)***	***198(47*.*75;171*.*25–219)***	***0*.*036***	133.5(69.5;110–179.5)	154(55.5;133.5–189)	0.71
**AT**	106(19;98–117)	93(3;90–93)	0.48	94.5(19.25;90.25–109.5)	98.5(23.75;91.5–115.25)	0.18

Variable values are represented as median (IQR; minimum-maximum values). To calculate the differences between the groups, we used the T test for normal distribution variables or the Mann-Whitney U test for variables without normal distribution. Abbreviations: TTPa = activated partial thromboplastin time; TP = prothrombin time; INR = international normal ratio; Fg = Fibrinogen; FvW = von Willebrand factor; D-D = D-dimers; AT = Antithrombin; D = detected; ND = not detected. Quantification values: aPTT (seconds); PT (% of activity); INR (ratio) Fg (mg / dL); vWF (% of activity); D-D- (ng / ml FEU); AT (% of activity).

Finally, we evaluated whether age would influence hemostatic parameters in SG and CG. For this purpose, we grouped each group into two subgroups: individuals aged 60–67 years and one aged 68–91 years. The data were then compared (Table 6).

**Table 6:** Biological parameters of hemostasis in elderly HIV reactive patients and control group, by age range.

	HIV+		Control		
Variable	A (n = 74)	B (n = 41)	C (n = 35)	D (n = 53)	CC (P *valor*)
	60–67 years old	68–91 years old	60–67 years old	68–87 years old	
**aPTT (seconds)**	28(3.98;26–29.98)	28.2(5.6;26.3–31.9)	29(3.77;26.8–30.57)	28.35(4.35;26.87–31.2)	NS
**PT (% of activity)**	93.9(16.4;85.6–102)	88.85(15.57;83.03–98.6)	90.9(14.4;85.6–100)	86.9(10.1;80.8–90.9)	A-B (0.046)
					C-B (0.003)
**INR (ratio)**	1.03(0.1;0.99–1.09	1.07(0.1;1.01–1.11)	1.05(0.09;1–1.1)	1.08(0.07;1.05–1.12)	C-B (0.001)
**Fg (mg/dL)**	387(126.25;324.25–450.5)	415(234;299–533)	406(121;343.5–464.5)	453(125.75;400–525.75)	C-D (0.042)
					A-D (0.003)
**D-D (ng/mL FEU)**	251(290;158–448)	345.5(434.25;186.5–620.75)	275(215.5;181–396.5)	503(529;319–848)	A-B (0.004)
					C-B (0.001)
					D-B (0.047)
**vWF (% of activity)**	155(56;127–183)	120.5(95.5;87.75–183.25)	131(48;108–156)	152(60;118–178)	NS
**AT (% of activity)**	105.5(24.25;92–116.25)	97(16;93–109)	89(12;85–97)	87(14.5;80–94.5)	A-C (0.003)
					C-B (<0.0001)
					D-A (0.001)
					D-B (<0.0001)

Variable values are represented as median(IQR;minimum-maximum value) using columns identifications by age range. In order to calculate differences between groups, a post-hoc Kruskal-Wallis and Dunn’s test was carried. P values < 0.05% were considered significant. Abbreviations: CC = columns compared; aPTT = activated partial thromboplastin time; PT = Prothrombin time; INR = International normalized ratio; Fg = Fibrinogen; vWF = von Willebrand factor; D-D = D-dimers; AT = Antithrombin; NS = not significant.

It was observed that HIV-reactive individuals aged 68–91 years (group B) more frequently incurred differences in hemostatic variables between groups. This older HIV group was characterized by having larger values of PT activity % when compared to the younger HIV^+^ group (60–67 years; *p* = 0.046) and control group (60–67 years, p = 0.003). It also had higher D-D values than younger groups (CG 60–67, *p* = 0.004; SG 60–6, *p* = 0.009) but lower than the older control group (68–91) (*p* = 0.047). Fibrinogen was increased in the older groups (*p* = 0.003; *p* = 0.042, respectively). The most significant differences were found in relation to AT. HIV-reactive individuals in both age groups had significantly higher values than those in the control group. Those in the older HIV group (68–91 years) had higher AT values when compared to the control group (*p*<0.0001 for each comparison).Similarly, HIV^+^ subjects aged 60–67 years had higher AT values when compared to control subjects (*p* = 0.003 and 0.001, respectively).

## Discussion

Viral HIV infections are characterized by acute and chronic inflammation, leading to critical cellular and protein changes that can modify coagulation biology. Here we study some recognized hemostatic parameters, typically altered in patients with HIV, and focus this assessment on an emerging and rarely studied population, chronically HIV-infected older people.

We compared the values of hemostatic variables between healthy individuals and those infected with HIV. We found higher values of AT and PT activity in HIV group compared with healthy controls. This result is consistent with previous observations in which hemostatic abnormalities were evaluated in untreated HIV-reactive men indifferent age groups and situations, showing significant values of altered proteins related to disease activity and its progression [28,49,56]. In a study with men with median of 44 years old, treated and untreated HIV-infected and uninfected control group was foundthat the median Antithrombin levels were higher while the endogenous thrombin potential, a functional measure of thrombin generation in vitro, was lower among HIV-infected adults (treated and untreated), compared with controls. They concluded that HIV infection was associated with decreased thrombin generation [57]. In another cross-sectional study of 66 HIV-infected individuals on antiretroviral treatment and undetectable viral load and 34 uninfected subjects, both elderly (>50 years old), no difference was found between groups regarding AT (25). Untreated HIV infection has also been shown to lead to a short-term decrease in AT, influencing thrombin generation [17]. This change has been suggested as HIV-related coagulation abnormalities that may be related to changes in liver function during inflammation in the acute phase. Our results are in accordance with the literature, for the approach as a disease outside the acute phase, however, having a differential that is the age group of the participants of these studies, which in others are observed in younger populations (average 45 years). There is an observed increase in AT in women with age and the opposite in men [54], but not observed in our analysis.

Due to haematological, hemorrhagic, and thrombotic clinical changes caused by HIV infection, coagulation tests such as PT / INR and aPTT are often considered as global hemostatic markers. A reduction in clotting time in these tests has been associated with hypercoagulability.PT, APTT, and INR were significantly higher, whereas platelet count was significantly lower in HIV-infected adults (both who were taking HAART and HAART-naïve) than HIV-seronegative adults (P<0.001) [58]. We do not know in the medical literature references about the normal range in the elderly or if the values are different from the general population.The exception is when they have vascular diseases such as hypertension and diabetes, use of heart prostheses, hepato-intestinal diseases. Likewise, in acute and chronic HIV infection and characterized by thrombotic complications with adverse vital tissue lesions [7,17,19,47,59]. Conflicting results have been observed in HIV infection mainly regarding the type of cohort in each study.For example, Omoregie et al evaluated 70 subjects and found greater PT and aPTT elongation in subjects with CD4^+^ lymphocyte count <200 / mm^3^ [60]. However, the epidemiological characteristics of the population studied were not defined. Nasir et al found no changes in TP among 128 HIV^+^ treated patients and individuals without HIV and the median age of the subjects studied was 35.7 years [61]. Considering the age differences and the predominance of the male population in these studies, we were unable to make a comparative analysis consistent with our results.

Next, we studied the influence of IP use, viral load and CD4^+^ considering by sex, the variables Fg, FvW, D-D and aPTT only in HIV-reactive individuals (Table 3). All subjects in this group were on ART and controlled disease.

It was observed that Fg was increased in individuals using protease inhibitors, regardless of sex. This change coincides with observations made by Jong et al who studied 160 subjects on ART. They found higher levels of Fg in subjects using PI compared to patients not using it [56]. As is most common, the study population consisted of 92% male individuals with a median age of 47 years and with comorbidities. It was not possible to directly compare our results with Jong's, due to the low percentage of females in his manuscript. Another study also showed an increase in Fg in younger HIV patients, mean age 43 years, on PI [62].

We observed in our cohort that the female group using PI had higher levels of vWF compared to the group without PI. Protease inhibitors are reported in the literature as agents that induce metabolic, bone, vascular, and cardiological changes while being involved in endothelial lesions, contributing to inflammatory reactions [63–65]. Inaddition, inflammation, monocyte activation, bacterial translocation, and lower viral load were observed in individuals undergoing PI monotherapy [66]. Here, all HIV^+^ subjects studied were on ART and 50% were on PI. Our results confirm the metabolic complications found in HIV^+^ subjects treated in other studies, such as dyslipidemia, with more than two years of IP use [4,65,67]. Fibrinogen is an acute phase protein and is considered an independent risk biomarker related to HIV infection and aging [56,64]. Sinha et al described microvascular and coagulation changes in a group of HIV^+^ patients composed mainly of men, 40% hypertensive and 25% smokers [16]. In their cohort, 78% of subjects were on PI. Chow et al found a two-fold increase in ischemic stroke in HIV-reactive women compared with healthy controls [18]. Similarly, HIV^+^ individuals on PI had higher Fg values than those in the control group, probably due to drug action [16,56,62]. Sinha et al described microvascular and coagulation changes in a group of HIV^+^ patients composed mainly of men, 40% hypertensive and 25% smokers [16]. In their cohort, 78% of individuals in use of PI. In our study, we found higher values of aPTT in the female group using IP, a fact not observed in our male group. It is suggested that PI may influence coagulation changes detected in menopause.The interaction of ART, in the case of PI, competing with female hepatic hormones in the use of P53 and estrogen deprivation with early menopause of these patients, are poorly studied topics, especially in the area of hemostasis [68]. Evidence of pre-thrombotic status was also reported in a multiethnic study that found an association between female sex hormones and changes in hemostatic variablesin general population [33]. Although hemostatic variables may also be related to early menopause, Bull et al pointed to difficulties in discriminating which factors trigger early menopause in HIV^+^ women [69]. Despite the importance of these results, attention is drawn to the low number of studies on hemostatic markers in postmenopausal women with ART-controlled infection. In fact, there is a warning about the need for studies focusing on HIV + female populations in this situation [70].

Regarding changes related to TCD4^+^ cell count, we observed higher values of vWF for the male HIV reactive group with > 400 cells / mm^3^. We know that lower TCD4^+^ T counts (<200 cells / mm^3^) are related to drastic changes in coagulation and disease progression [60,61,71]. In studies with younger populations, the change in vWF was associated with changes in inflammatory and coagulation markers [57,71]. In our study, we had only a small number of patients with <200 TCD4^+^cells / mm^3^ (n = 5), which did not allow further comparisons with these results. In addition, most patients were on treatment, stable in their disease (with 97 of 115 patients with undetectable serum viral load and 88 subjects). Some hemostatic parameters that remain altered are probably due to subclinical infections and should be analyzed in conjunction with viral load [60,71]. In the present study, the viral load detected had a low influence on hemostatic variables for SG, composed of twelve women and six men. Among the SG individuals, only the female group presented alterations in vWF. Some reports have associated menopause, viral load and endothelial dysfunction with increased vWF [55,72,73]. Elevations in vWF can be observed in disease progression studies, including HIV infection, without being related to gender [55]. Also our vWF results should be carefully considered given the small sample size of the male group with> 400 CD4^+^ T cells / mm^3^. In a systematic review of inflammatory and immunological changes, was hypothesized that these vWF changes were related to estrogen deprivation [74]. Menopause has also been suggested as a low-grade systemic inflammation due to changes in the peripheral immune system [73]. When studying the association between menopause and aging, changes in hemostatic proteins were reported (higher Fg, FvW, AT, among others, in all coagulation phases [68]. Overall, our results showed that undetectable viral load and CD4^+^ T counts (most patients had counts > 200 cells / mm^3^) could explain the low number of changes found in our study.

Due to the successful treatment of HIV, infected individuals have a longer life expectancy (over 60 years of age). Studies on the effect of aging on hemostatic variables are not frequent. Age is a predisposing factor for a higher incidence of cardiovascular, neurocognitive and thrombotic events. Studies involving D-D, Fg, vWF, and CD4^+^T cell values have associated antiretroviral treatment of HIV infection with normalization of previously altered indicators [25,34]. A prospective multicenter cohort of individuals over 50 years of age with HIV^+^ reported a slow immune recovery from this population compared to younger subjects, both on ART [75]. Alternatively, a study of individuals with chronic stage HIV over the age of 50 described that hemostatic factors could serve as predictors of neurocognitive imbalance in these patients [25].

To assess whether age could influence changes in hemostatic variables, we divided individuals according to age groups (60–67 years) and (68–91 years) into the two groups. We found several differences in values between groups associated with SG, especially in the group 60–67 years. There was a difference between the HIV ^+^ groups in the PT values.We did not find references in the literature about these data. The PT activity was within normal values in both groups and the value (*p* = 0.046) little statistical impact, which was reflected in the non-significant INR values. Age reflected in the comparative results of the groups in relation to Fibrinogen values, especially in the CG and between the younger SG (60–67 years) and the older CG (68–87 years). Fibrinogen increases with age, and this result is consistent with those in the literature [22,27,76]. The D-D values in the SG had a significant difference in relation to age group (*p* = 0.004). In CG there was no difference in this variable. There was a difference between the older groups (SG x CG), with higher values for the CG, but with little impact (*p* = 0.047). D-D are independent factors in aging and their comorbidities [42,77]. The statistical age difference in the HIV^+^ group in relation to the D-D could be explained by the biological early aging in this population [21], which is not the case with the CG [45]. The vWF values did not show statistical differences between the groups with different ages, disagreeing with their reported increase in aging [35] and, in our case, in patients with HIV under controlled disease [55].

Antithrombim values in all groups were within normal reference ranges, with higher medians in the HIV + group. In this group there was no difference between the two age groups studied, as there were no differences between the control group. The younger HIV (60–67 years) and older HIV (68–91 years) groups showed differences with the control group. This finding is more consistent with the status of the infection than with age. A study on coagulation changes in young and elderly individuals (without HIV infection) found a decrease in AT and an increase in Fg in older individuals, with greater impact on females [76]. Another study showed an increase in AT in women and a decrease in men with aging [54]. Our cohort consisted of male and female subjects, and during the evaluation of variables such as viral load, CD4^+^ cell counts and protease inhibitor use, AT showed no differences according to gender. Increased AT has been shown in treated or untreated HIV patients compared to negative controls in younger individuals [57]. Our results suggest that age had no influence on this variable.

Regarding natural aging, numerous studies on changes in hemostasis have been described [27,35,39,40,78]. Reports of these changes in the elderly have similarities to those found in patients with chronic HIV infection [17,55,57]. Inflammatory aging status is suggested in HIV-reactive patients before infection progression [79–81]. Aging is an important component in the mortality of the HIV reactive population, observed mainly in some sub-Saharan African populations [6,10]. Inflammation and cellular changes in aging and immunosenescence are associated with prothrombotic status in HIV^+^ patients [24,61]. Genetic conditions, mitochondrial DNA alterations and telomerase activity, lifestyle, hypertension and diabetes also influence the markers of these conditions [22]. HIV infection and aging have been studied on many fronts, led by these well-known markers. The involvement of coagulopathy in HIV infection is related to the severity of inflammation and associated with aging as well, providing new perspectives on the clinical and laboratory understanding of HIV pathogenesis [82]. In this scenario, despite the role of hemostasis in the pathophysiology of HIV, its impact is not yet clear in this specific population.

To our knowledge, this is one of the first studies describing hemostatic changes in HIV^+^ individuals aged > 60 years on ART, with low frequency of aging complications. Here, we found significant differences in Antithrombin and Prothrombin Time in HIV^+^ subjects compared to negative controls. The greater increase in antithrombin appears to be associated with successful treatment, particularly in younger HIV patients, as opposed to pre-thrombotic status, a condition found in HIV infection. Age, viral load and use of protease inhibitors influenced hemostatic variables when we analyzed the HIV group in particular. Specifically, the use of protease inhibitors altered outcomes in HIV^+^ women by more than one parameter, probably due to menopausal interference. Overall, we assume that a better hemostatic balance in HIV^+^ patients found in our study may be related to controlled disease status and better personal care than those found in uninfected individuals. Finally, these data need to be evaluated in the context of other related associations, such as inflammatory markers, as well as other components of it, such as the role of microbiome composition, in order to better explain the coagulation-inflammation-aging process in infection with HIV. This study needs validation with larger cohorts.
