# Correction: Hemostasis in elderly patients with human immunodeficiency virus (HIV) infection—Cross-sectional study

**DOI:** 10.1371/journal.pone.0230727

**Published:** 2020-03-16

**Authors:** Marilza Campos de Magalhães, Juan Camilo Sánchez-Arcila, Ana Carolina de Brito Lyra, Luiz Felipe Boufleur Long, Isabelle Vasconcellos de Souza, Fernando Raphael de Almeida Ferry, Adilson José de Almeida, Soniza Vieira Alves-Leon

There are errors in the Author Contributions. The correct contributions are:

**Conceptualization:** Marilza Campos de Magalhães, Fernando Raphael de Almeida Ferry

**Data curation:** Ana Carolina de Britto Lyra, Luiz Felipe Boufleur Long, Isabelle Vasconcelos de Souza

**Formal analysis:** Juan Camilo Sanchez-Árcila, Isabelle Vasconcellos de Souza

**Funding acquisition:** Fernando Raphael de Almeida Ferry

**Investigation**: Ana Carolina de Britto Lyra, Luiz Felipe Boufleur Long, Isabelle Vasconcelos de Souza

**Methodology:** Marilza Campos de Magalhães, Adilson José da Almeida, Juan Camilo Sanchez-Árcila, Soniza Vieira Alves-Leon

**Project administration:** Marilza Campos de Magalhães

**Resources:** Fernando Raphael de Almeida Ferry

**Supervision:** Juan Camilo Sanchez-Árcila, Soniza Vieira Alves-Leon

**Visualization:** Juan Camilo Sanchez-Árcila, Soniza Vieira Alves-Leon

**Writing–original draft:** Marilza Campos de Magalhães

**Writing–review & editing:** Marilza Campos de Magalhães, Juan Camilo Sanchez-Árcila
